# Berberine ameliorates thioacetamide-induced liver injury in zebrafish through PPARγ signaling and restores bile acid homeostasis

**DOI:** 10.3389/fphar.2026.1881966

**Published:** 2026-10-12

**Authors:** Peng Sun, Jun Wu, Xiaoyu Zhu, Yanshuang Ren, Zhanyi Zhou, Wenjian Zhen, Yuan Ma, Xue Ding, Menghan Huang, Yuying Zhang, Wei Gong, Hao Wang

**Affiliations:** 1 Science and Technology Center, Ningxia Medical University, Yinchuan, China; 2 Ningxia Hui Autonomous Region Institute of Medical Sciences, Yinchuan, China; 3 School of Public Health, Ningxia Medical University, Yinchuan, China; 4 Key Laboratory of Environmental Factors and Chronic Disease Control, Ningxia Medical University, Yinchuan, China; 5 The First Clinical Medical College, Ningxia Medical University, Yinchuan, China; 6 School of Pharmacy, Ningxia Medical University, Yinchuan, China; 7 School of Basic, Ningxia Medical University, Yinchuan, China; 8 Medical Experiment Center, General Hospital of Ningxia Medical University, Yinchuan, China; 9 School of Medical Information and Engineering, Ningxia Medical University, Yinchuan, China

**Keywords:** berberine, bile acid metabolism, *Coptis chinensis*, nonalcoholic fatty liver disease, PPARγ pathway, zebrafish

## Abstract

**Ethnopharmacological Relevance:**

*Coptis chinensis* Franch. (Huanglian), a traditional Chinese medicinal herb used for “heat” and “dampness” syndromes, is clinically associated with metabolic disorders such as NAFLD. Berberine (BBR), its major bioactive alkaloid, has shown metabolic benefits, though its mechanistic basis in NAFLD remains unclear.

**Aim of the Study:**

To identify candidate pathways underlying the effects of BBR in NAFLD, a computational target prediction approach was employed, followed by *in vivo* experimental validation in a zebrafish model.

**Materials and Methods:**

A thioacetamide (TAA)-induced liver injury zebrafish model was established. Hepatic lipid accumulation and histopathology were assessed by Oil Red O staining, H&E staining, and qPCR. A computational target prediction approach was employed to identify potential BBR-NAFLD overlapping targets, followed by pathway enrichment analysis and molecular docking simulations to prioritize candidate pathways. Bile acid profiles were analyzed using UHPLC-MS/MS metabolomics. The predicted PPARγ pathway was experimentally validated using the specific antagonist GW9662 and the agonist rosiglitazone.

**Results:**

BBR significantly reduced hepatic steatosis and restored liver morphology. A total of 104 overlapping targets were identified, with PPAR signaling emerging as a key pathway. BBR showed stable binding to PPARγ (−7.92 kcal/mol). *In vivo,* BBR restored the expression of pparg, rxra, cyp7a1, cyp27a1, and cyp8b1, all of which were suppressed by TAA; these effects were reversed by GW9662, confirming PPARγ dependence. Metabolomics revealed reduced levels of 6-ketoLCA and UDCA-3S, which were partially reversed by PPARγ inhibition.

**Conclusion:**

BBR alleviates TAA-induced liver injury by regulating PPARγ and bile acid homeostasis, integrating systems pharmacology and experimental validation to support the ethnopharmacological use of *C. chinensis* in metabolic liver disease.

## 1 Introduction

Non-alcoholic fatty liver disease (NAFLD) is the most prevalent chronic liver disorder worldwide, affecting approximately 25% of adults (Ahmed et al., 2015; Cotter and Rinella, 2020), and its prevalence in China has increased rapidly to nearly 30% (Lu et al., 2023). In addition to progressive hepatic injury, NAFLD is strongly associated with extrahepatic metabolic complications (Marengo et al., 2016; Powell et al., 2021; Muthiah et al., 2022), leading to substantial socioeconomic burden, reduced quality of life, and increased mortality. Despite extensive research efforts, the multifactorial and heterogeneous pathogenesis of NAFLD has hindered effective drug development, and no approved pharmacotherapy is currently available (Friedman et al., 2018). Therefore, identifying novel therapeutic agents and elucidating their underlying mechanisms remain urgent priorities in hepatology.

*Coptis chinensis* Franch., commonly known as Huanglian, is a classical herb with a clinical history spanning over two millennia. Its dried rhizome has been widely used since the Han dynasty, as documented in the *Shennong Bencao Jing*, and it remains a key component in more than 32,000 traditional prescriptions (Wang et al., 2019). In Traditional Chinese medicine (TCM) theory, Huanglian is characterized as bitter in taste and cold in nature, with actions attributed to the heart, liver, spleen, stomach, gallbladder, and large intestine meridians. It is traditionally used to clear heat, dry dampness, purge fire, and detoxify (Singh et al., 2024). Importantly, these traditional indications closely correspond to TCM syndrome patterns frequently observed in NAFLD, particularly “damp-heat accumulation in the liver and spleen” and “phlegm retention due to spleen deficiency”, providing a strong ethnopharmacological rationale for its application in metabolic liver disorders (Sun et al., 2024). Modern pharmacological studies have further confirmed that *C. chinensis* exhibits a broad spectrum of bioactivities, including anti-inflammatory, antioxidant, antidiabetic, hypolipidemic, hepatoprotective, antimicrobial, and anticancer effects (Singh et al., 2024).

The principal bioactive metabolite of *C. chinensis* is berberine (BBR), an isoquinoline alkaloid that has attracted considerable attention due to its broad therapeutic potential. Both clinical and preclinical studies have demonstrated that BBR ameliorates multiple pathological features of NAFLD. When combined with lifestyle interventions, BBR significantly improves body weight, insulin resistance (HOMA-IR), serum lipid profiles, and hepatic steatosis (Huang et al., 2023; Jia et al., 2024; Nie et al., 2024). In experimental models, BBR administration restores gut microbiota homeostasis and attenuates hepatic fat accumulation in mice fed a high-fat, high-sucrose diet (Hang et al., 2025; Yin et al., 2026). *In vitro* studies further revealed that BBR attenuated fructose-induced injury in HepG2 cells (Cheng et al., 2024; Khaled et al., 2024). Although BBR has a significant beneficial effect on NAFLD, its specific role and underlying mechanism in NAFLD still need to be fully elucidated.

In recent years, integrative strategies combining systems pharmacology and multi-omics approaches have provided new theoretical frameworks and technical avenues for elucidating the mechanisms of complex traditional medicines (Tan et al., 2024; Zhai et al., 2025). Unlike the conventional single-target research paradigm, network analysis coupled with metabolomics analysis enables multi-level dissection of the holistic regulatory networks underlying drug actions. This comprehensive approach not only facilitates the identification of key regulatory nodes and core signaling axes but also provides verifiable molecular evidence to support traditional medicinal practices. Zebrafish (*Danio rerio*) has emerged as a valuable *in vivo* model for investigating hepatic and metabolic disorders due to its high genetic and physiological similarity to humans. Juvenile zebrafish models of NAFLD are well established and widely used as a cost-effective platform for screening potential therapeutics (Goessling and Sadler, 2015; Ma et al., 2019; An et al., 2021).

Although dietary models, such as high-fat or high-cholesterol diets, more closely reproduce the metabolic disturbances characteristic of human NAFLD (Jin et al., 2024; Bhattacharya et al., 2025), chemically induced zebrafish models offer practical advantages for early-stage drug screening, including rapid phenotype development, good reproducibility, and suitability for mechanistic studies (Asaoka et al., 2013). Previous studies have shown that TAA exposure can induce hepatic lipid accumulation, hepatomegaly, oxidative stress, and inflammatory responses in zebrafish larvae (Amali et al., 2006; Rekha et al., 2008; Cao et al., 2025). The TAA-induced zebrafish model has also been used to evaluate the hepatoprotective effects of candidate compounds against hepatic steatosis and liver injury (Hammes et al., 2012). Accordingly, we used the TAA-induced model as an initial *in vivo* screening model to assess the protective effects of BBR on hepatic lipid.

In this study, we combined network analysis, molecular docking, molecular dynamics simulations, and *in vivo* validation to investigate the potential mechanisms underlying the protective effects of BBR. This integrated approach allowed us to identify potential molecular targets and signaling pathways and to further examine their relevance *in vivo*. Our findings provide new insights into the hepatoprotective effects of BBR and may help clarify the pharmacological basis of *C. chinensis* in the regulation of hepatic lipid, providing a basis for further investigation of its therapeutic potential in NAFLD.

## 2 Materials and methods

### 2.1 Materials

Wild-type AB strain zebrafish (*Danio rerio*) were obtained from the China Zebrafish Resource Center (Wuhan, China) and maintained under standard laboratory conditions. Berberine (BBR; Cat. No. B414323, purity ≥99%) was purchased from Shanghai Aladdin Biochemical Technology Co., Ltd. Thioacetamide (TAA; Cat. No. A600940). The MightyScript™ Reverse Transcription Kit (Cat. No. B639251) and the 2× Universal Blue SYBR Green qPCR Kit (Cat. No. G1015) were obtained from BBI Life Sciences (Shanghai, China). Saturated Oil Red O staining solution was purchased from Wuhan Servicebio Technology Co., Ltd. All other chemicals used were of analytical grade unless otherwise specified.

### 2.2 Zebrafish husbandry

Zebrafish were maintained at 28.5 °C ± 1 °C under a 14 h light/10 h dark cycle in a recirculating aquatic system. Adult fish (5 months old) were fed twice daily with freshly hatched *Artemia nauplii*. For breeding, healthy adults were paired at a 2:1 male-to-female ratio. Males and females were separated the day before mating and subsequently placed together in breeding tanks overnight. Fertilized eggs were collected the following morning and examined at 3 h post-fertilization (hpf) under a stereomicroscope (M205C, Leica, Germany) to confirm viability. Embryos were incubated at 28.5 °C in embryo medium composed of 5 mM NaCl, 0.17 mM KCl, 0.33 mM CaCl_2_, and 0.33 mM MgSO_4_ (pH 7.2) under standard conditions.

### 2.3 NAFLD model induction and drug treatment

At 3 days post-fertilization (dpf), morphologically normal zebrafish larvae were randomly allocated into six experimental groups: (1) control group (system water only for 48 h); (2) TAA model group (7 mM TAA for 48 h); (3) TAA + BBR group (7 mM TAA for 48 h, with 25 μM BBR added during the last 24 h); (4) TAA + GW9662 group (7 mM TAA for 48 h, with 5 μM GW9662 added during the last 24 h); (5) TAA + BBR + GW9662 group (7 mM TAA for 48 h, with 25 μM BBR and 5 μM GW9662 added during the last 24 h); and (6) TAA + rosiglitazone group (7 mM TAA for 48 h, with 50 nM rosiglitazone added during the last 24 h).

For histological analysis (Oil Red O and H&E staining), each group contained 30 individual larvae (n = 30). For all biochemical (TG and TC, serum ALT and AST), molecular (qPCR), and metabolomic (UHPLC-MS/MS) assays, larvae were pooled as biological replicates using whole-body homogenates, with each pool consisting of 30 larvae collected from one well of 6-well plates; three such pools (n = 3 pools) were prepared per group (Supplementary Figure S4). This pooling strategy has been validated in previous zebrafish metabolomics studies (van Mever et al., 2023; Min et al., 2024).

Larvae in all TAA-exposed groups were treated with 7 mM thioacetamide for 48 h to induce hepatic steatosis and liver injury. The TAA concentration was selected based on a previously established zebrafish acute liver injury model (Deng et al., 2025). For drug intervention, BBR (25 μM(Chen et al., 2019)), GW9662 (5 μM(Jin et al., 2020), a specific PPARγ antagonist), and rosiglitazone (50 nM(Tiefenbach et al., 2010; Tiefenbach et al., 2018), a classical PPARγ agonist) were added to the zebrafish medium starting 24 h after TAA exposure and continuing for an additional 24 h. The BBR concentration was selected based on a previous study demonstrating that 25 μM BBR significantly prevented hepatic steatosis in zebrafish larvae. The GW9662 concentration was selected based on previous zebrafish studies demonstrating effective PPARγ inhibition at 5 μM. Rosiglitazone was dissolved in DMSO and diluted with embryo medium to a final concentration of 50 nM, with the final DMSO concentration not exceeding 0.1% (v/v) in any treatment group. An equivalent amount of DMSO was added to the control and model groups. All experiments were performed in duplicate under identical environmental conditions.

### 2.4 Evaluation of hepatic steatosis and histopathology

Hepatic lipid accumulation was evaluated using Oil Red O staining, while liver tissue morphology was assessed via histological analysis. Larvae were first rinsed with phosphate-buffered saline (PBS) and then fixed before staining with Oil Red O for 3 h, followed by destaining in 60% isopropanol. Lipid deposition was quantified using stereomicroscopic imaging, with 20 biological replicates per group. For histopathological analysis, fixed larvae were embedded in paraffin, sectioned at 4 µm thickness, and stained with hematoxylin and eosin (H&E). Sections were examined under a light microscope to evaluate hepatic architecture and pathological alterations.

### 2.5 Drug-likeness and toxicity profiling of BBR

The drug-likeness and toxicity profiles of BBR were evaluated using SwissADME (http://www.swissadme.ch/) and ADMETlab 2.0 (https://admetmesh.scbdd.com/). Computational assessments were conducted in accordance with Lipinski’s Rule of Five, with the following thresholds: molecular weight <500 Da, hydrogen bond acceptors <10, hydrogen bond donors <5, Moriguchi octanol-water partition coefficient (LogP) < 4.15, and topological polar surface area <140 Å^2^. Comprehensive ADMET profiling, including absorption, distribution, metabolism, excretion, and toxicity, was performed using ADMETlab 2.0 to predict BBR’s pharmacokinetic properties and safety profile. The analysis emphasized BBR’s potential to cross physiological barriers *in vivo*, aiming to optimize bioavailability and minimize toxicity. Key toxicity endpoints assessed included hERG channel inhibition, human hepatotoxicity, ocular irritation, respiratory toxicity, and acute oral toxicity in rats.

### 2.6 Identification and screening of BBR targets

The putative molecular targets of BBR were identified through an integrated database mining approach utilizing the following resources: TCMSP (https://old.tcmsp-e.com/tcmsp.php), HERB (http://herb.ac.cn/), ChEMBL (https://www.ebi.ac.uk/chembl/), DrugBank (https://go.drugbank.com/), and TTD (https://db.idrblab.net/ttd/). The canonical three-dimensional structure of BBR (CID 2353) was retrieved from PubChem (https://pubchem.ncbi.nlm.nih.gov/).

Target prediction was further performed using the PharMapper database (http://www.lilab-ecust.cn/pharmmapper/) and Swisstargetprediction database (http://www.swisstargetprediction.ch/). Following data collection, we performed deduplication of target entries and standardized protein nomenclature by converting all identifiers to official gene symbols through UniProt (https://www.uniprot.org/).

### 2.7 Identification and screening of NAFLD targets

Targets associated with NAFLD were retrieved from multiple databases, including GeneCards (https://www.genecards.org/), Therapeutic Target Database (TTD, https://db.idrblab.net/ttd/), Online Mendelian Inheritance in Man (OMIM, https://www.omim.org/), UniProt (https://www.uniprot.org/), and DrugBank (https://go.drugbank.com/), using the keyword “Non-alcoholic fatty liver disease”. After data collection, duplicate entries were removed to ensure consistency and accuracy of the dataset.

### 2.8 Identification of BBR-NAFLD common targets

Overlapping targets between BBR and NAFLD were identified using Venny 2.1.0 (https://bioinfogp.cnb.csic.es/tools/venny/). The overlapping genes were defined as common BBR-NAFLD targets and were used for subsequent PPI network and functional enrichment analyses.

### 2.9 PPI network construction and topological analysis

The common BBR–NAFLD targets were submitted to the STRING database (version 11.5; https://cn.string-db.org/) to construct a protein–protein interaction (PPI) network. The species was restricted to *Homo sapiens*, and the minimum required interaction score was set to 0.700. Nodes without interaction partners were excluded. The PPI network was imported into Cytoscape 3.9.1.

### 2.10 GO and KEGG pathway enrichment analysis

Gene Ontology (GO) and Kyoto Encyclopedia of Genes and Genomes (KEGG) pathway enrichment analyses were performed on the overlapping targets of BBR and NAFLD using the DAVID online database (v6.8; http://www.david.ncifcrf.gov) in combination with KEGG pathway mapping. GO enrichment encompassed three ontological categories: biological process (BP), cellular component (CC), and molecular function (MF). Statistical significance was set at *P* < 0.05, with Benjamini-Hochberg adjusted *P* values (false discovery rate, FDR) < 0.05. For visualization, the top ten significantly enriched terms were plotted using the Bioinformatics online platform (http://www.bioinformatics.com.cn).

### 2.11 Functional module analysis

Functional modules within the PPI network were analyzed using Metascape (https://metascape.org/) and the Molecular Complex Detection (MCODE) algorithm. MCODE was used to identify densely connected clusters within the PPI network. Modules containing at least three proteins were retained for further analysis. Functional enrichment analysis was subsequently performed for the identified modules, and terms with Benjamini-Hochberg-adjusted *P* < 0.05 were considered significant.

### 2.12 Integrated screening of candidate targets and pathways

Candidate targets and pathways were prioritized from the common BBR–NAFLD targets by integrating PPI network topology, KEGG pathway enrichment, MCODE functional module analysis, and their relevance to hepatic lipid metabolism. For target prioritization, the topological characteristics of nodes in the PPI network were considered together with the distribution of targets across significantly enriched pathways and MCODE modules. Pathways related to lipid metabolism, fatty acid metabolism, bile acid metabolism, and hepatic steatosis were given greater consideration based on their biological relevance to liver metabolic disorders. Targets showing relatively high network centrality and involvement in the prioritized pathways or functional modules were considered candidate targets for further investigation. Candidate targets and pathways identified through this integrated analysis were subsequently evaluated by molecular docking and experimental validation.

### 2.13 Construction of the BBR-target-pathway-NAFLD network

The BBR-target-pathway-NAFLD network was constructed using Cytoscape 3.9.1. Candidate targets identified by the integrated screening procedure and the top 20 significantly enriched KEGG pathways were included in the network. BBR, NAFLD, candidate targets, and pathways were represented as nodes, and their corresponding relationships were represented as edges. Node size and color were adjusted according to the degree value to visualize the connectivity of each node.

### 2.14 Molecular docking

Molecular docking was performed to predict the potential therapeutic effects of BBR by evaluating the binding affinities between active compounds and key protein targets. Docking simulations were carried out using Schrödinger Suite 2023 (version 13.5). The 3D structure of berberine was obtained in Mol2 format from the TCMSP database. Two core target proteins were selected from the RCSB Protein Data Bank (https://www.rcsb.org/), filtered by species “*Homo sapiens*” and the selection criterion of “Unique Ligands” using the minimum ligand-based method. Protein structures were preprocessed using the Protein Preparation Wizard in Schrödinger, which included tasks such as assignment of bond orders, addition of hydrogen atoms, removal of water molecules beyond 5 Å from the active site, and restrained energy minimization. Receptor grids were subsequently generated based on the coordinates of native ligands within the active sites. Ligand docking was performed using Glide (version 6.5) in Standard Precision (SP) mode. The docking scores were used to estimate binding affinity, and compound–protein interaction modes were visualized and analyzed using Maestro (version 2023).

### 2.15 Molecular dynamics (MD) simulation

Molecular dynamics (MD) simulations of the BBR-PPARγ complex were performed using the Desmond module in Schrödinger Suite 2023. The protein–ligand complex obtained from molecular docking was prepared using the Protein Preparation Wizard and solvated in an orthorhombic box with the TIP3P explicit water model. The OPLS3e force field was used, and Na^+^/Cl^−^ ions were added to neutralize the system and maintain physiological ionic strength. The system was subjected to energy minimization and equilibration under NVT and NPT ensembles according to the standard Desmond protocol (Cai et al., 2023). A production simulation was subsequently performed for 100 ns under constant temperature and pressure conditions. The resulting trajectories were analyzed using the Simulation Interaction Diagram module. Root-mean-square deviation (RMSD), Root-mean-square fluctuation (RMSF), radius of gyration (Rg), hydrogen bonds, and protein-ligand interactions were analyzed to assess the stability and conformational changes of the BBR-PPARγ complex.

### 2.16 RT-qPCR analysis

Total RNA was extracted from zebrafish liver tissues using the MightyScript™ Reverse Transcription Kit (BBI Life Sciences), following the manufacturer’s instructions. cDNA was synthesized and amplified using the 2×Universal Blue SYBR Green qPCR Kit, with glyceraldehyde-3-phosphate dehydrogenase (GAPDH) as the internal reference gene. Primer sequences were listed in Supplementary Table S1. Relative gene expression levels were calculated using the 2^−ΔΔCt method.

### 2.17 Targeted metabolomic analysis

Targeted bile acid profiling was performed using an ultra-high-performance liquid chromatography-tandem mass spectrometry (UHPLC-MS/MS) system (ExionLC™ AD UHPLC coupled with QTRAP® 6500+, AB SCIEX, Boston, MA, United States). Seventy-two bile acid standards and six stable isotope-labeled internal standards were obtained from ZZ Standards Co., Ltd. (Shanghai, China). Analytical-grade ammonium acetate was purchased from Sigma-Aldrich (St. Louis, MO, United States), and LC-MS grade methanol, acetonitrile, and formic acid were obtained from Thermo Fisher Scientific (FairLawn, NJ, United States).

Chromatographic separation was performed on a Waters ACQUITY UPLC BEH C18 column (2.1 × 100 mm, 1.7 μm) maintained at 50 °C. The mobile phase consisted of 0.1% formic acid and 5 mM ammonium acetate in water (solvent A) and acetonitrile (solvent B), delivered at 0.35 mL/min. The gradient program was as follows: 5% B at 0–0.5 min, 5%–30% B at 0.5–2.0 min, 30%–37% B at 2.0–6.0 min, 37%–38% B at 6.0–7.0 min, 38%–39% B at 7.0–7.5 min, 39%–42% B at 7.5–8.0 min, 42%–43% B at 8.0–8.5 min, 43%–50% B at 8.5–12.0 min, 50%–60% B at 12.0–13.0 min, 60%–95% B at 13.0–14.0 min, 95%–5% B at 14.0–14.5 min, and re-equilibration at 5% B until 15 min.

Mass spectrometric detection was performed in negative multiple reaction monitoring (MRM) mode. The instrument parameters were set as follows: IonSpray voltage, −4500 V; curtain gas, 30 psi; source temperature, 550 °C; and ion source gases 1 and 2, 60 psi. Data acquisition and quantification were conducted using Analyst software (AB SCIEX).

### 2.18 Statistical analysis

All experimental data are presented as mean ± standard deviation (SD). Statistical analyses were performed using GraphPad Prism version 8.0 (GraphPad Software, San Diego, CA, United States). Comparisons between two groups were conducted using unpaired Student’s t-test, while multiple group comparisons were performed using one-way analysis of variance (ANOVA) followed by Tukey’s *post hoc* test. Differences were considered statistically significant at *P* < 0.05.

## 3 Results

### 3.1 BBR treatment attenuates TAA-induced liver injury in zebrafish larvae

To determine the optimal concentration of BBR, TAA-treated zebrafish larvae were exposed to BBR at 1, 5, 25, or 50 μM. Oil Red O staining showed a progressive reduction in TAA-induced hepatic lipid accumulation with increasing BBR concentrations up to 25 μM, whereas no additional improvement was observed at 50 μM (Supplementary Figure S1). Consistent with the staining results, 25 μM BBR showed the strongest effects on reducing TG and TC levels and ALT and AST activities among the tested concentrations (Supplementary Figure S2). qPCR analysis further showed that 25 μM BBR significantly reversed the TAA-induced upregulation of pparg and rxra mRNA expression, while concurrently upregulating the mRNA levels of cyp7a1, cyp8b1, and cyp27a1, which encode key enzymes involved in bile acid synthesis (Supplementary Figure S3). Therefore, 25 μM BBR was used in the subsequent mechanistic experiments.

We next evaluated the therapeutic effect of 25 μM BBR on TAA-induced liver injury, healthy zebrafish larvae at 3 dpf were exposed to 7 mM TAA) for 24 h to induce hepatic steatosis. Subsequently, larvae in the intervention group were treated with 25 μM BBR for an additional 24 h (Figure 1A). TAA-exposed larvae exhibited pronounced hepatomegaly, darkened liver pigmentation, and extensive intrahepatic lipid accumulation, including prominent lipid deposition in the caudal vasculature, intestinal veins, and intersegmental vessels. BBR treatment significantly alleviated these pathological changes, reducing hepatic steatosis and improving vascular lipid clearance (Figure 1B). Histological analysis further revealed that TAA exposure resulted in marked disruption of hepatic architecture, including cytoplasmic vacuolization and loss of parenchymal integrity, consistent with advanced NAFLD pathology. In contrast, BBR-treated larvae displayed markedly improved liver morphology, with restored tissue organization, decreased vacuolization, and reduced lipid infiltration (Figure 1C).

**FIGURE 1:**
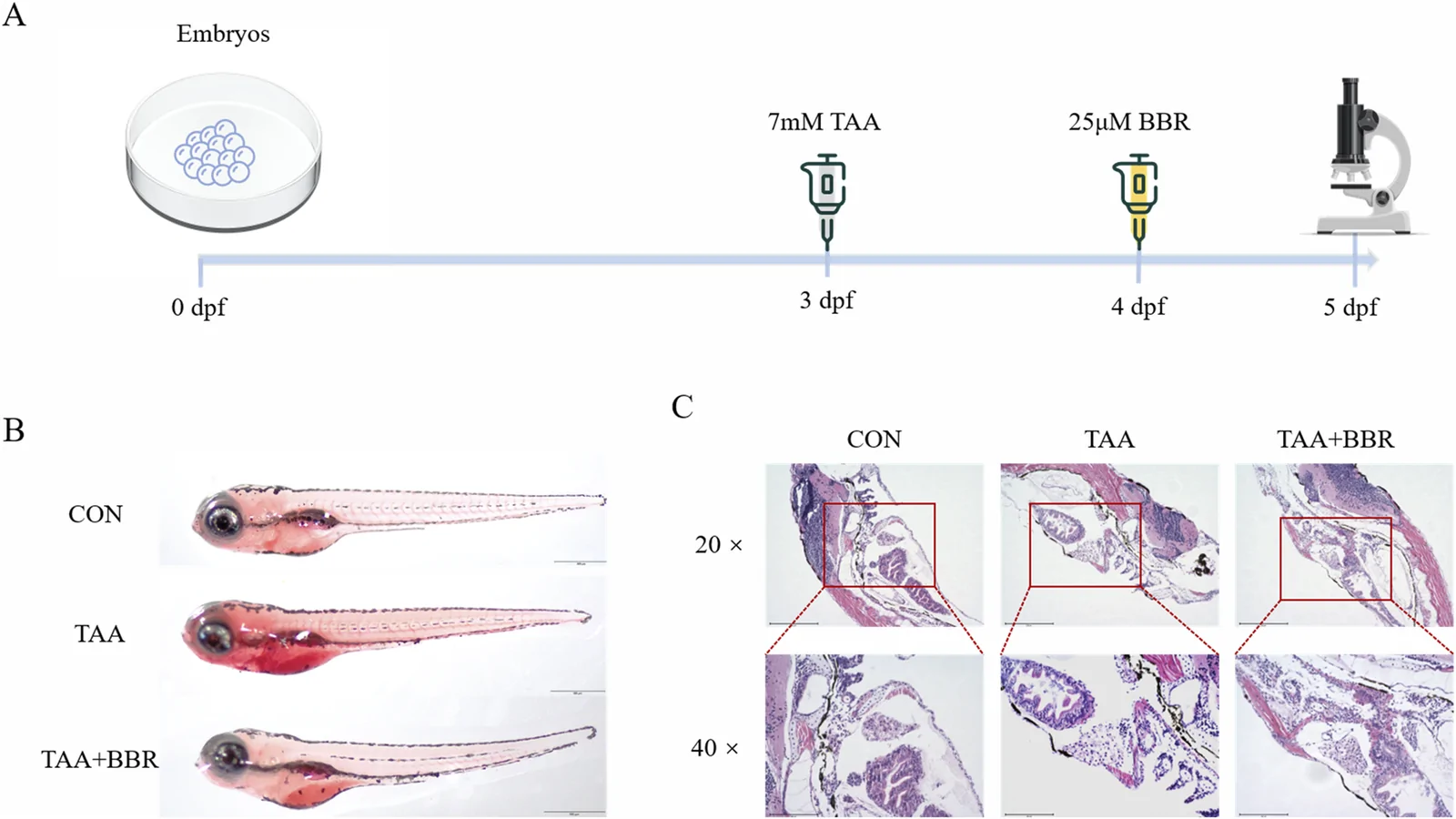
BBR intervention improved TAA-induced hepatic steatosis and liver injury in zebrafish. **(A)** Experimental workflow. **(B)** Representative images of Oil Red O staining (mean ± SD, n = 30). **(C)** Representative images of H&E staining (mean ± SD, n = 30).

### 3.2 Network analysis predicts mechanism of BBR in NAFLD

#### 3.2.1 BBR drug-likeness and toxicity analysis

The pharmacokinetic properties, drug-likeness, and toxicity profile of BBR were evaluated using SwissADME and ADMETlab 2.0. BBR showed high predicted gastrointestinal absorption and fully complied with Lipinski’s Rule of Five, with acceptable molecular weight, hydrogen bond donor/acceptor counts, and lipophilicity parameters (Table 1). Toxicological predictions indicated a relatively low risk of hERG channel inhibition, human hepatotoxicity, ocular irritation, and acute oral toxicity in rats. However, BBR showed a potential risk of respiratory toxicity in the predicted pulmonary toxicity assessment (Table 2).

**TABLE 1:** BBR Drug Similarity Analysis

Compound	Structure	MW	MLogP	Nhbd	nHBA	TPSA	Absorption
		(≤ 500Da)	(< 5)	(≤ 5)	(≤ 10)	(< 140Å2)	
BBR		336.36	2.19	0	5	40.8	high

**TABLE 2:** BBR Drug Toxicity Analysis

Parameter	hERG Blockers	Human hepatotoxicity	Eye Corrosion	Respiratory Toxicity	Rat Oral Acute Toxicity
BBR	Non-blocker	Negative	Negative	Positive	Negative

#### 3.2.2 BBR target prediction

Potential molecular targets of BBR were systematically identified using multiple databases, including TCMSP, HERB, ChEMBL, DrugBank, TTD, PharmMapper, and SwissTargetPrediction. The number of targets retrieved from each database was 17, 339, 2, 21, 3, 100, and 213, respectively (Supplementary Table S2). Following removal of duplicate entries and non-human targets, a total of 302 unique BBR-related targets were retained for subsequent network analysis (Supplementary Table S3).

#### 3.2.3 NAFLD target prediction

NAFLD-associated targets were retrieved from multiple databases, including GeneCards, OMIM, DrugBank, DisGeNET, and TTD. After filtering and removal of duplicate entries, the number of NAFLD-related targets obtained from each database was 1340, 482, 4, 26, and 1, respectively, yielding a total of 1853 unique targets (Supplementary Table S4).

#### 3.2.4 Selection of overlapping targets between BBR and NAFLD

To identify potential therapeutic targets of BBR in NAFLD, the intersection between 302 BBR-related targets and 1853 NAFLD-related targets was determined using Venny 2.1.0 (https://bioinfogp.cnb.csic.es/tools/venny/). A total of 104 overlapping targets were identified, representing putative molecular targets through which BBR may exert its anti-NAFLD effects (Figure 2A; Supplementary Table S5).

**FIGURE 2:**
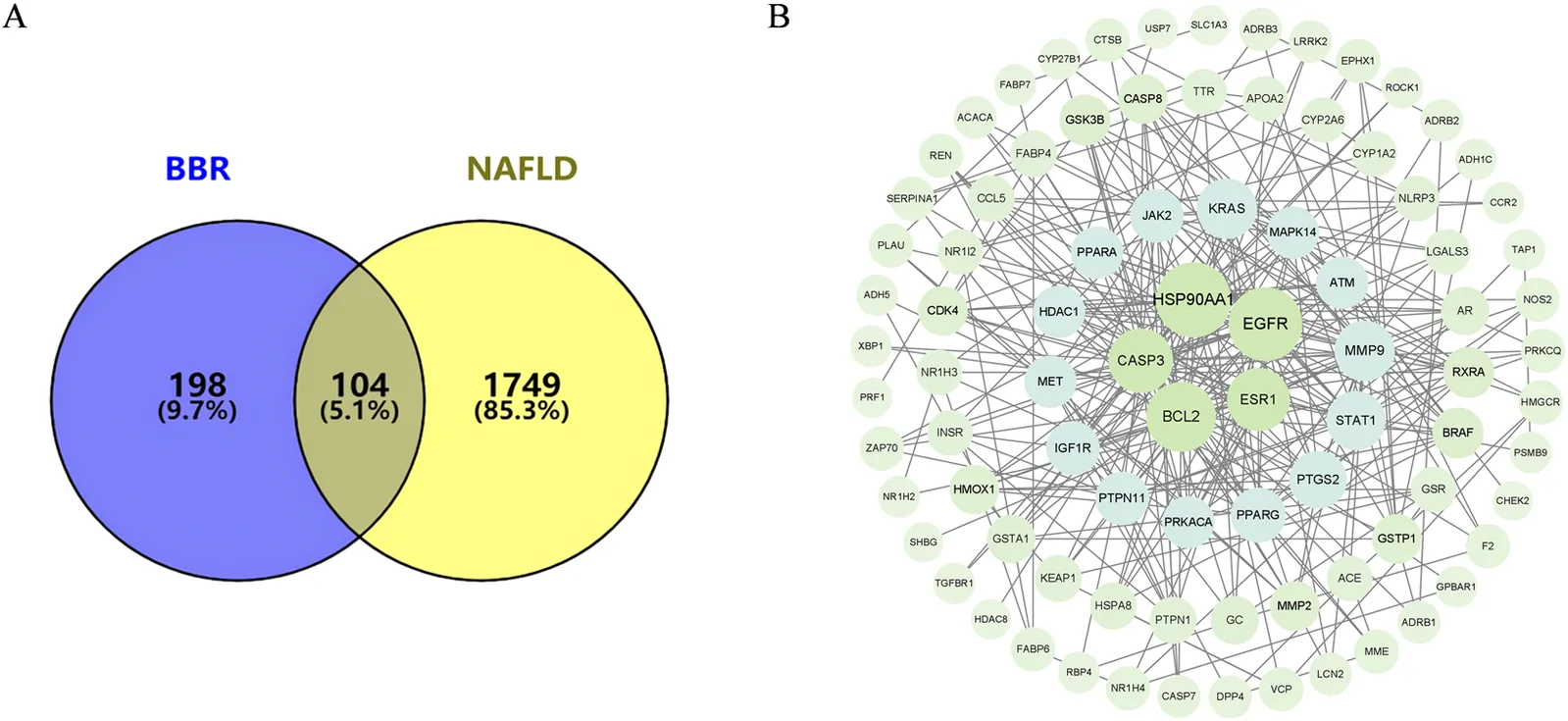
Screening of BBR in NAFLD. **(A)** Venn diagram of intersecting target genes of BBR and NAFLD. **(B)** Protein-protein interaction network.

#### 3.2.5 PPI network construction and core target screening

The 104 overlapping targets were imported into the STRING database (version 11.5; https://cn.string-db.org/) to construct a PPI network. The minimum required interaction confidence score was set at 0.700, and disconnected nodes were removed to enhance network reliability. Topological analysis was performed using Cytoscape (version 3.9.1) with the CytoNCA plugin, and closeness centrality was adopted as the primary screening metric. The resulting protein-protein interaction network consisted of 104 nodes representing proteins and 283 edges representing interactions between them (Figure 2B; Supplementary Table S6). Node sizes were scaled according to their degree centrality, reflecting the relative importance and connectivity of each protein within the network.

#### 3.2.6 PPI network construction

The 104 overlapping BBR-NAFLD targets were submitted to the STRING database to construct a PPI network. *Homo sapiens* was selected as the reference organism, and the minimum interaction confidence score was set to 0.700. Disconnected nodes were excluded. The resulting PPI network contained 104 nodes and 283 edges (Figure 2B; Supplementary Table S6). Several proteins showed relatively dense interactions within the network, including HSP90AA1, CASP3, BCL2, ESR1, EGFR, PPARγ, and STAT1. The PPI network was imported into Cytoscape 3.9.1 for visualization and subsequent functional module analysis.

#### 3.2.7 GO and KEGG pathway enrichment analysis and integrated prioritization of candidate targets

GO and KEGG pathway enrichment analyses were performed using the DAVID platform on the 104 overlapping targets of BBR and NAFLD. GO analysis identified 395 significant entries, including 272 in BP, 41 in CC, and 82 in MF. Enriched BP terms included positive regulation of cell proliferation, gene expression, response to estradiol, transcription regulation by RNA polymerase I promoter, and protein phosphorylation. CC terms were mainly associated with extracellular matrix, cell surface, myofibril, and platelet alpha granule lumen, while MF terms encompassed protein binding, growth factor activity, RNA polymerase II transcription factor activity, and sequence-specific DNA binding activated by ligands. The top 10 significant GO terms (*P* < 0.05) were presented in Figure 3A and Supplementary Table S7. KEGG pathway enrichment analysis identified 123 significantly enriched pathways. Several pathways related to metabolic regulation and liver-related pathological processes were significantly enriched, including the PPAR signaling pathway, IL-17 signaling pathway, and AGE-RAGE signaling pathway. The top 20 enriched pathways are presented in Figure 3B and Supplementary Table S8. The PPAR signaling pathway was considered of particular interest because of its established involvement in lipid metabolism, fatty acid metabolism, and hepatic steatosis. The targets involved in this pathway are shown in Figure 3C. To further identify core therapeutic modules, the MCODE algorithm was applied to construct a modular network from the overlapping targets. Topological analysis revealed that the principal pathways involved included pathways in cancer, chemical carcinogenesis-receptor activation, lipid and atherosclerosis, IL-17 signaling pathway, AGE-RAGE signaling pathway in diabetic complications, and the PPAR signaling pathway (Figure 3D). To clarify target-pathway relationships, a subset network was generated (Figure 3E), enabling visualization of target functions across different clusters. Comprehensive MCODE clustering using Metascape identified five common target modules for BBR in NAFLD treatment (Figure 3F; Supplementary Table S9). Candidate targets and pathways were selected based on the KEGG enrichment results, MCODE analysis, and their known roles in hepatic lipid metabolism and steatosis. The PPAR signaling pathway was of particular interest because of its involvement in lipid metabolism and hepatic steatosis. PPARG, a key component of this pathway, was selected for subsequent molecular docking and experimental validation.

**FIGURE 3:**
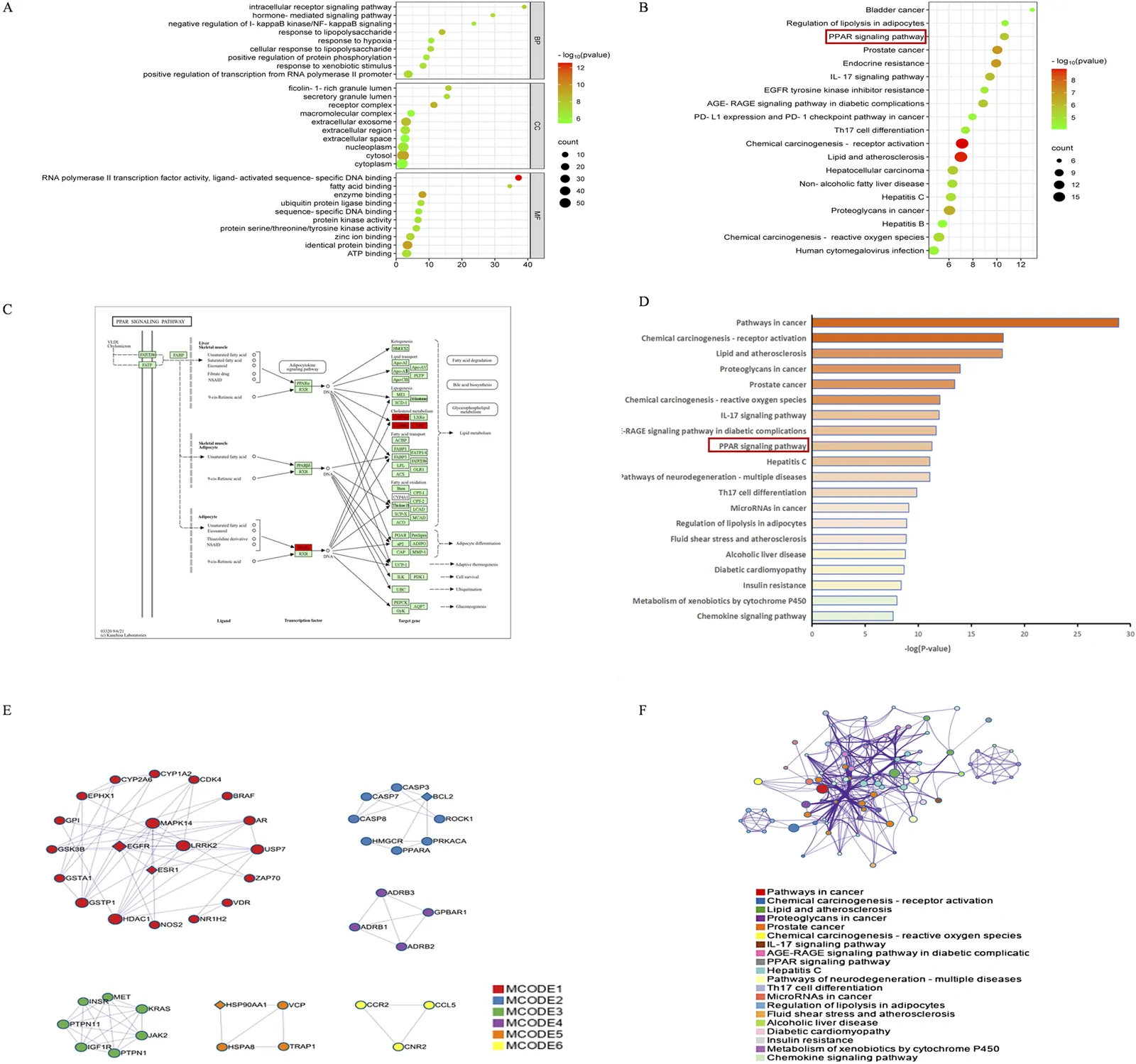
GO, KEGG pathway enrichment analysis and cluster module analysis diagram of related protein targets of 104 overlapping genes associated with BBR and NAFLD. **(A)** GO analysis of BBR and NAFLD. **(B)** KEGG pathway enrichment of BBR and NAFLD. **(C)** PPAR signaling pathway. **(D)** Highly enriched terms of BBR in NAFLD. **(E)** Sub network specific to the interaction. **(F)** Cluster analysis of BBR in NAFLD.

#### 3.2.8 Construction of the “target-pathway-disease” network

To visualize the interactions between BBR, NAFLD-related targets, and enriched signaling pathways, a “NAFLD-BBR-target-pathway” network was constructed using Cytoscape 3.9.1. The network consisted of 127 nodes and 362 edges (Figure 4), where nodes represent targets, bioactive components, or pathways, and edges indicate interactions between them. In the network diagram, BBR was represented by a purple hexagon, NAFLD by an orange V-shaped node, pathways by orange hexagons, and overlapping BBR-NAFLD target genes by green circles. Node size and color intensity correspond to degree centrality, reflecting the relative importance of each node within the network. The top 12 targets, ranked by degree, were identified as key hub proteins and listed in Supplementary Table S10.

**FIGURE 4:**
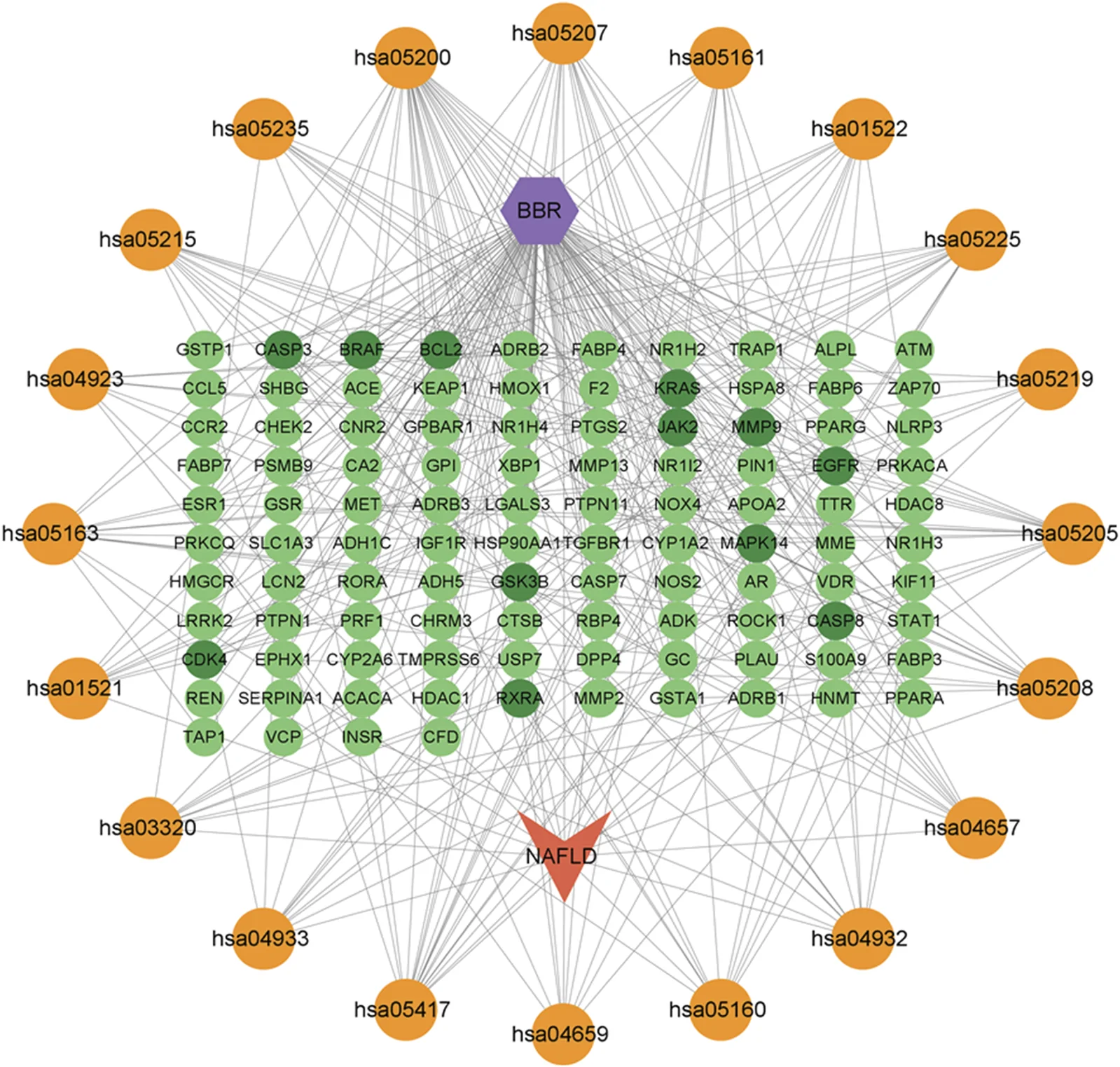
Component-target-pathway diagram of BBR in NAFLD.

### 3.3 Experimental evaluation of the potential role of PPARγ

#### 3.3.1 Molecular docking and molecular dynamics analysis

Molecular docking was performed to examine the potential interaction between BBR and PPARγ. PPARγ was selected based on the results of the network analysis and its established role in hepatic lipid metabolism. BBR showed a docking score of −7.92 kcal/mol with PPARγ (Figure 5A), while rosiglitazone, a well-established PPARγ agonist, showed a score of −7.057 kcal/mol under the same docking conditions (Supplementary Figure S4). The docking results indicated a favorable predicted interaction between BBR and PPARγ. MD simulation was subsequently performed using Desmond. The RMSD remained relatively stable throughout the simulation, indicating that the BBR-PPARγ complex was maintained during the simulation period (Figure 5B). Most PPARγ residues showed relatively low RMSF values, with no marked fluctuations observed in the major regions of the protein (Figure 5C). Together, the docking and MD results support a potential interaction between BBR and PPARγ, although these computational findings do not by themselves demonstrate direct activation of PPARγ.

**FIGURE 5:**
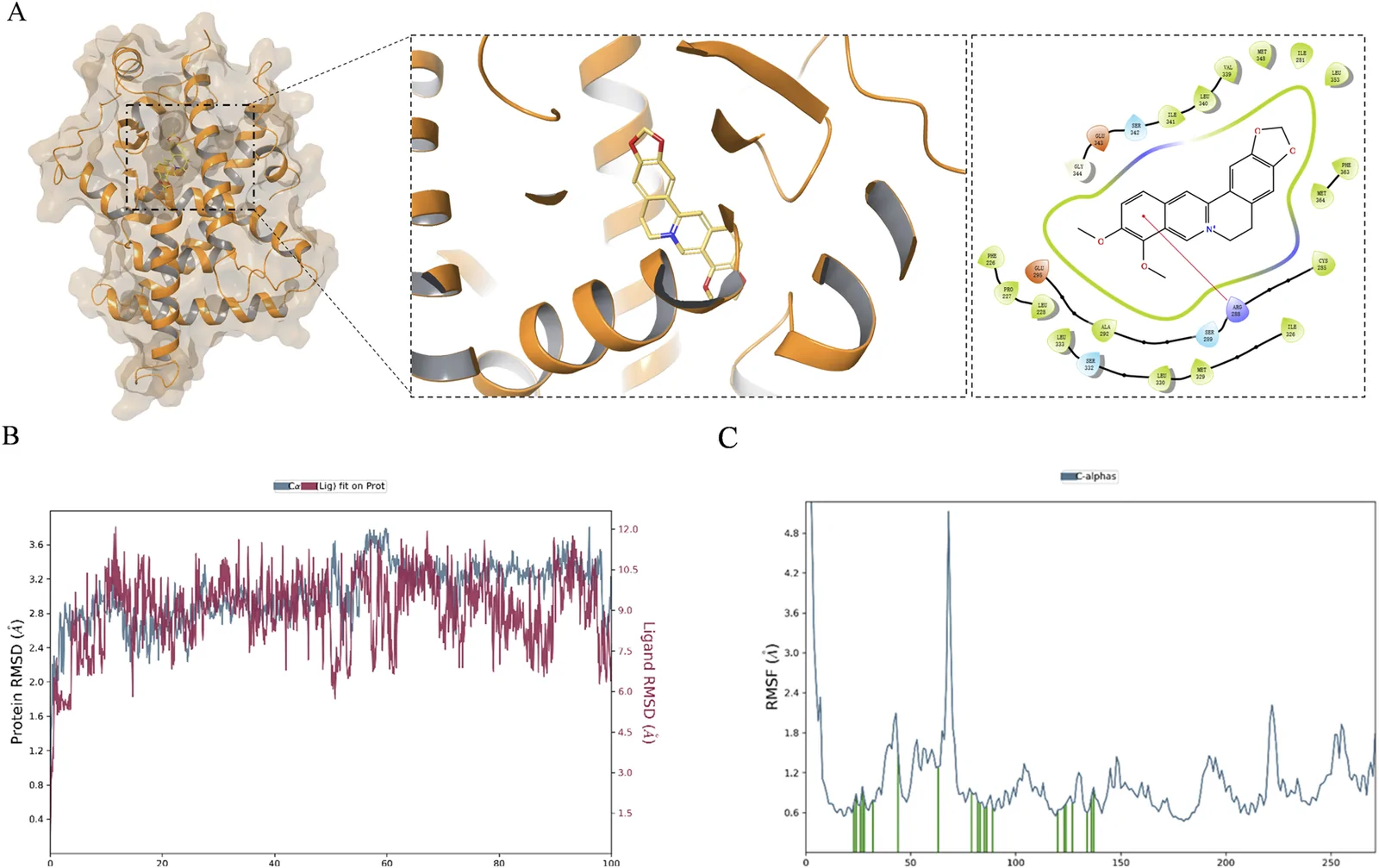
Molecular docking demonstration and MD Simulation of BBR with PPARγ and LXRα. **(A)** Docking demonstration of BBR with PPARγ. **(B)** RMSD fluctuation plot of the BBR-PPARγ complex. **(C)** RMSF fluctuation plot of the BBR-PPARγ complex. Residues in contact with the ligand are labeled in green.

#### 3.3.2 Effects of BBR on TAA-induced liver injury and PPARγ-associated changes in zebrafish

To validate the involvement of PPARγ in BBR-mediated hepatoprotection, we employed a TAA-induced zebrafish liver injury model using the optimal BBR concentration (25 μM), with or without co-treatment with the PPARγ-specific inhibitor GW9662 (5 μM), and compared with the PPARγ agonist rosiglitazone (50 nM) as a positive control.

Oil Red O staining revealed pronounced hepatic lipid accumulation in the TAA model group compared with controls. BBR (25 μM) markedly reduced lipid deposition, whereas co-treatment with GW9662 significantly attenuated BBR’s protective effects, resulting in the reappearance of severe lipid accumulation (Figure 6A). Notably, the TAA + GW9662-alone group showed no significant difference in lipid accumulation or biochemical parameters (TG, TC, ALT, AST) compared with the TAA model group, excluding non-specific effects of the inhibitor (Figures 6B–E). In contrast, the positive control group treated with rosiglitazone exhibited similar protective effects to BBR, further supporting the role of PPARγ in mediating the therapeutic response.

**FIGURE 6:**
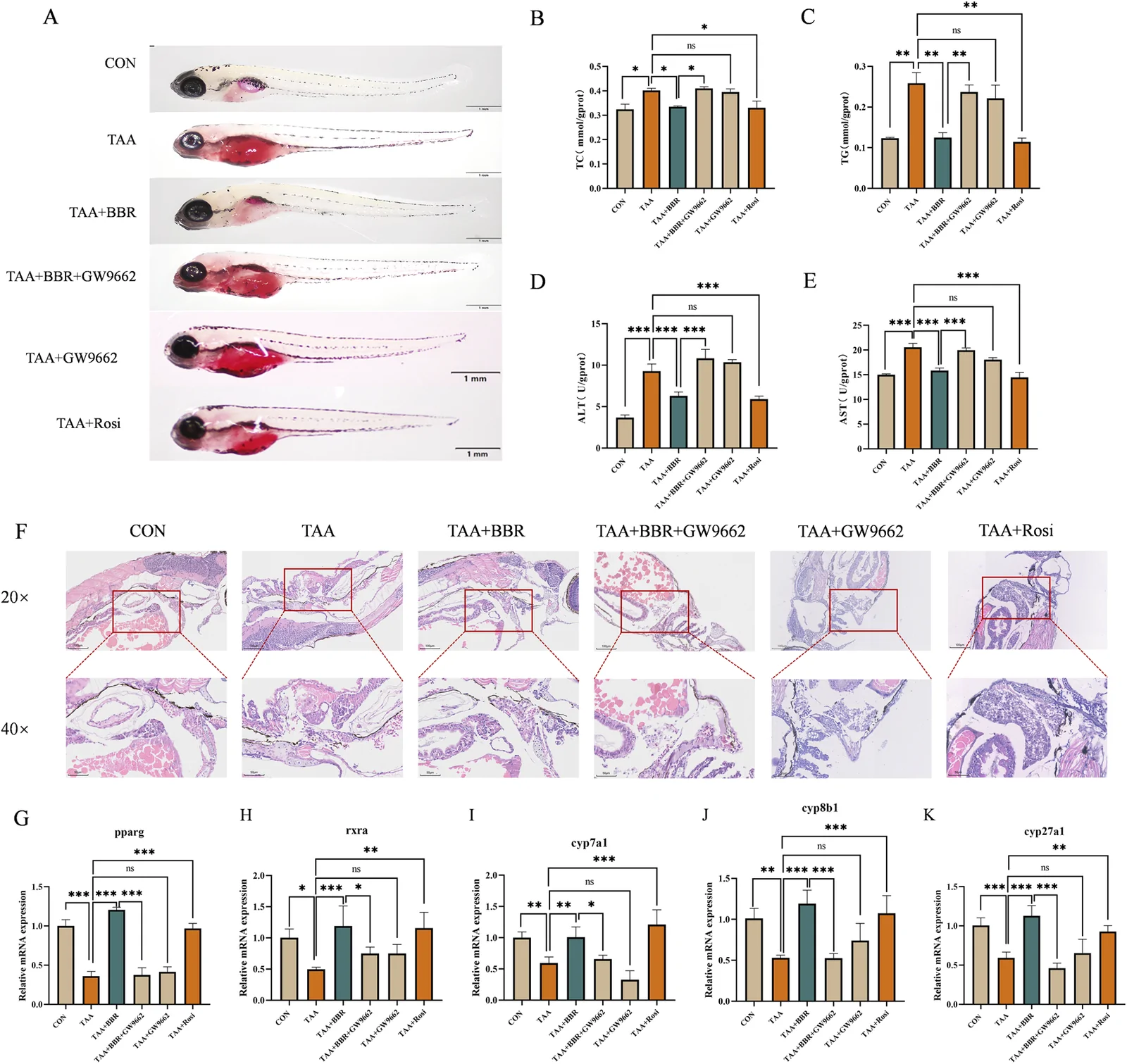
Effects of BBR on TAA-induced hepatic injury and PPARy-associated changes in zebrafish. **(A)** Representative images of Oil Red O staining showing hepatic lipid accumulation. **(B–E)** Biochemical parameters measured in whole-body homogenates: **(B)** TC, **(C)** TG, **(D)** ALT, and **(E)** AST **(F)** Representative images of H&E staining showing hepatic histopathological changes. **(G–K)** Relative mRNA expression levels of PPARy-associated genes determined by quantitative real-time PCR: **(G)** PPARG, **(H)** RXRA, **(I)** CYP7A1, **(J)** CYP27A1, and **(K)** CYP8B1. Data are presented as mean ± SD. For Oil Red O and histological analyses, n = 30 per group; biochemical and qPCR data are presented from three independent biological replicates. *P < 0.05, **P < 0.01, ***P < 0.001; ns, not significant.

Histopathological analysis using H&E staining demonstrated that TAA exposure caused disorganized hepatic architecture, hepatocyte vacuolar degeneration, and inflammatory cell infiltration. BBR treatment substantially ameliorated these pathological changes, restoring hepatocyte morphology and liver tissue organization. Notably, co-treatment with GW9662 partially reversed the beneficial effects of BBR, whereas rosiglitazone treatment mirrored the protective effects of BBR. These findings indicate that PPARγ is critical for BBR-mediated hepatoprotection (Figure 6F).

Real-time quantitative PCR analysis was performed to assess mRNA expression of key components in the PPARγ signaling pathway. TAA significantly suppressed the expression of pparg, rxra, and bile acid-synthesizing enzymes (cyp7a1, cyp27a1, cyp8b1). BBR at 25 μM effectively restored the expression levels of these genes. Rosiglitazone treatment similarly upregulated these genes, consistent with PPARγ pathway engagement. The protective effects of BBR were markedly inhibited by GW9662, confirming the dependency of BBR’s effects on PPARγ-mediated pathways (Figures 6G–K).

#### 3.3.3 Effects of BBR on bile acid profiles in TAA-induced zebrafish liver injury

To further elucidate the impact of BBR on bile acid metabolism, targeted bile acid metabolomics was performed using UHPLC-MS/MS. Multivariate statistical analysis, including principal component analysis (PCA), revealed clear separation among control, TAA model, TAA + BBR, and TAA + BBR + GW9662 groups, indicating that different treatments significantly altered the bile acid metabolic profiles (Figure 7A). Samples within each group exhibited tight clustering, confirming experimental reproducibility.

**FIGURE 7:**
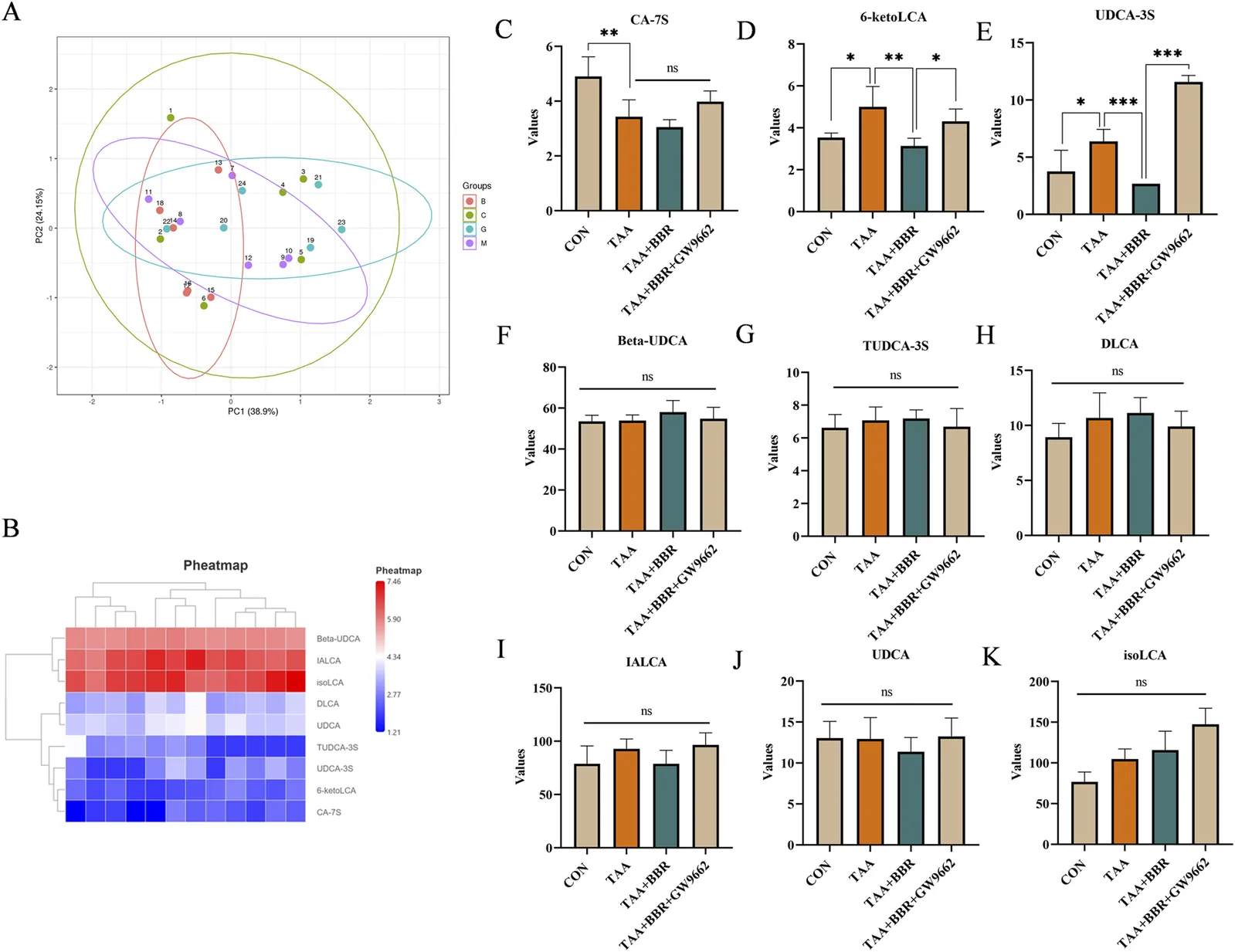
Metabolomic analysis of bile acids in zebrafish across groups. **(A)** Scatter plot obtained from principal component analysis (PCA). **(B)** Hierarchical clustering heatmap of differentially expressed bile acids. **(C–K)** Quantitative comparison of key bile acids. Data are presented as mean ± SD. *P < 0.05, **P < 0.01, ***P < 0.001; ns, not significant.

Hierarchical clustering analysis of nine differentially expressed bile acids demonstrated distinct patterns among groups (Figure 7B). Specifically, TAA exposure led to elevated levels of 6-ketoLCA and UDCA-3S and reduced levels of CA-7S, reflecting bile acid dysregulation induced by hepatic injury. BBR intervention significantly reversed the elevation of 6-ketoLCA and UDCA-3S, while CA-7S levels showed minimal recovery. Co-treatment with GW9662 partially abrogated BBR’s regulatory effects on 6-ketoLCA and UDCA-3S, indicating that their modulation occurs in a PPARγ-dependent manner (Figures 7C–E). Other bile acids, including β-UDCA, TUDCA-3S, DLCA, IALCA, UDCA, and isoLCA, exhibited no significant differences among the groups, suggesting that BBR selectively modulates specific bile acid metabolites associated with TAA-induced hepatic injury (Figures 7F–K). These findings highlight the role of PPARγ-mediated regulation in BBR’s hepatoprotective mechanism and support the functional relevance of the PPARγ axis in bile acid homeostasis. Overall, targeted metabolomic analysis demonstrated that BBR ameliorated TAA-induced disturbances in specific bile acid metabolites, particularly 6-ketoLCA and UDCA-3S, through a PPARγ-dependent pathway, consistent with the network analysis and molecular docking predictions.

## 4 Discussion

NAFLD is a multifactorial metabolic disorder characterized by hepatic lipid accumulation, insulin resistance, chronic inflammation, oxidative stress, and gut microbiota dysbiosis (Chang et al., 2010; Yang et al., 2011; Xu et al., 2019; Yang et al., 2024). Despite increasing interest in pharmacological interventions, no targeted therapy has yet achieved clinical approval. Traditional Chinese medicine (TCM) offers promising therapeutic avenues, with BBR, the main isoquinoline alkaloid of *C. chinensis*, attracting attention due to its hepatoprotective and lipid-lowering properties (Cai et al., 2023).

In this study, we employed an integrative approach combining network analysis, molecular docking, MD simulations, and *in vivo* validation to elucidate the mechanisms by which BBR ameliorates NAFLD. Using a TAA-induced zebrafish liver injury model, BBR effectively reduced hepatic lipid accumulation and improved liver morphology, indicating its protective effects against chemically induced metabolic liver injury, consistent with previous reports demonstrating its capacity to restore lipid homeostasis and attenuate liver injury (Yang et al., 2011; Xu et al., 2019; Yang et al., 2024). However, the TAA model does not fully recapitulate the metabolic features of human NAFLD, as TAA primarily causes chemical hepatocellular injury with secondary lipid accumulation (Asaoka et al., 2013). Thus, the TAA-induced zebrafish model used in the present study is more appropriate for the rapid evaluation of hepatoprotective effects against hepatic steatosis and acute liver injury than for modeling the full metabolic pathogenesis of NAFLD (Li et al., 2025; Walsh et al., 2025). Further studies using diet-induced NAFLD models and mammalian models will be needed to determine whether the protective effects of BBR observed in this study can be reproduced under conditions that more closely resemble human NAFLD. The chosen BBR concentration (25 μM) was based on prior studies showing efficacy without toxicity in zebrafish models (Jia et al., 2018).

While network analysis and molecular docking are useful for hypothesis generation, they are prone to false-positive predictions, particularly for compounds such as berberine, a known pan-assay interference compound (Nasiri et al., 2017; Bolz et al., 2021). Experimental evidence indicates that berberine can interfere with assay readouts and generate false signals in biochemical screening (Nasiri et al., 2017). Moreover, the databases used for target prediction are often interlinked, which may propagate biases (Diao et al., 2026). A systematic analysis of network-based studies has also highlighted a pattern of “homogeneity,” where similar molecules and pathways are repeatedly identified across diverse conditions, raising concerns about specificity (Diao et al., 2026). Therefore, in the present study, computational predictions were used exclusively to prioritize candidate pathways for experimental testing, while all mechanistic conclusions are grounded in the *in vivo* validation data.

Consistent with the computational predictions, the PPAR signaling pathway emerged as a candidate for BBR-mediated lipid regulation. PPARγ forms heterodimers with RXRα, coordinately regulating fatty acid oxidation, cholesterol efflux, and bile acid metabolism (Tyagi et al., 2011). Specifically, BBR restored the expression of cyp7a1, cyp27a1, and cyp8b1, all of which were downregulated by TAA exposure, suggesting a normalization of the bile acid synthetic profile, which governs bile acid hydrophobicity. These changes may promote the formation of less hepatotoxic bile acid species, reducing lipid-induced liver damage (Jahan and Chiang, 2005; Chevre et al., 2018; Jia et al., 2018; Jia et al., 2020). Molecular docking and MD simulations supported a potential interaction between BBR and PPARγ. The docking score of BBR was comparable to that of rosiglitazone, a well-established PPARγ agonist, and the BBR-PPARγ complex remained relatively stable during the MD simulation. These computational findings support further investigation of the role of PPARγ in the effects of BBR. It should be noted that network ana and molecular docking analyses were performed using human protein targets, while experimental validation was conducted in zebrafish. This cross-species approach is justified by the high evolutionary conservation of PPARγ and related CYP enzymes between humans and zebrafish. Zebrafish PPARγ shares conserved structural and functional domains with human PPARG, and its involvement in lipid metabolism regulation has been well characterized (Den Broeder et al., 2015). Similarly, CYP enzymes involved in bile acid synthesis and metabolism, including CYP7A1, CYP27A1, and CYP8B1, exhibit conserved biological functions across vertebrates (Loerracher and Braunbeck, 2021). Thus, the zebrafish model provides a suitable *in vivo* system for investigating the regulatory effects of BBR on PPARγ-associated metabolic pathways. Nevertheless, species-specific differences in metabolic regulation cannot be excluded, and further studies in mammalian models are needed to validate the involvement of PPARγ-associated signaling in the protective effects of BBR and to assess the translational relevance of these findings.

Targeted bile acid metabolomics further demonstrated that TAA treatment led to significant increases in 6-ketoLCA and UDCA-3S, a significant decrease in CA-3S, and no significant changes in the remaining six bile acids. 6-ketoLCA is a hydrophobic secondary bile acid that has been reported to be elevated in NAFLD patients and associated with liver injury, serving as a potential biomarker of hepatic damage (Ferslew et al., 2015; Liu et al., 2023). Sulfation, catalyzed by cytosolic sulfotransferases, is a major detoxification pathway that increases bile acid polarity and solubility to facilitate excretion and prevent cholestatic injury (Saini et al., 2004; Alnouti, 2009; Larabi et al., 2023); the elevation of UDCA-3S, a sulfated conjugate of UDCA, thus likely reflects a sulfation-mediated compensatory clearance response to bile acid accumulation (Alnouti, 2009). Following BBR intervention, both 6-ketoLCA and UDCA-3S decreased significantly in a synchronized manner, and GW9662 completely blocked this effect, demonstrating that BBR downregulates these molecules through restoration of PPARγ-mediated signaling, consistent with the known role of PPARγ in regulating bile acid synthesis and transport via genes expression including CYP7A1, CYP27A1, and multiple transporters (Wang et al., 2018; He et al., 2025). Unlike these two molecules, the TAA-induced decrease in CA-3S was not reversed by BBR nor affected by GW9662, indicating PPARγ-independent regulation, likely through FXR or LXR (Liu et al., 2022). Collectively, these findings suggest that, under the present experimental conditions of BBR intervention in the TAA-induced model, 6-ketoLCA and UDCA-3S may serve as pharmacodynamic indicators reflecting the activation of PPARγ signaling, consistent with the emerging role of bile acid profiles as indicators of metabolic liver disease (Liu et al., 2023; He et al., 2025).

We acknowledge the paradoxical nature of PPARγ modulation in hepatic steatosis. Full PPARγ agonists such as thiazolidinediones typically promote lipid accumulation in hepatocytes, and hepatic PPARγ activation may even attenuate the therapeutic potential of these drugs in NAFLD by exacerbating steatosis (Lee et al., 2023). In our TAA-induced liver injury model, PPARγ expression was significantly suppressed by TAA, and BBR restored its expression to near-normal levels. This pattern is consistent with a restorative modulation, rather than a direct agonism, as we did not observe PPARγ overexpression beyond baseline levels. Importantly, BBR treatment upregulated the expression of bile acid synthetic enzymes (CYP7A1, CYP27A1, CYP8B1) and reduced the levels of 6-ketoLCA and UDCA-3S, suggesting pathway-selective modulation. GW9662-mediated partial reversal of BBR’s effects confirms PPARγ dependence of these actions. However, the present data do not establish that BBR directly binds to or activates PPARγ as a classical agonist. Future studies using PPARγ reporter assays and target gene profiling are warranted to clarify the precise mode of BBR-PPARγ interaction. Although the network analysis was performed using human databases, the conserved functions of PPARγ and several bile acid metabolism-related enzymes between mammals and zebrafish support the use of zebrafish for preliminary *in vivo* validation. Nevertheless, species-specific differences in metabolic regulation cannot be excluded. Further studies in mammalian models are needed to validate the involvement of PPARγ-associated signaling in the protective effects of BBR and to assess the translational relevance of these findings.

Several limitations should be acknowledged. First, the TAA model represents chemical injury-induced steatosis rather than metabolic NAFLD, and findings should be validated in diet-induced or genetically modified models to enhance translational relevance; the optimal dose and long-term safety of BBR in mammals also require further investigation. Second, while the network analysis predictions and PPARγ restoration observed in our study suggest a functional link, the direct binding of BBR to PPARγ and the direct transcriptional regulation of CYP7A1 by PPARγ remain to be definitively established, and further studies using SPR, ChIP qPCR, and PPRE-driven luciferase reporter assays are needed to establish the causal chain. Third, the zebrafish-specific bile acid C27-cholic acid was not detected, and its interaction with C24-cholic acids remains unclear. Further studies are warranted to clarify the metabolic crosstalk between C27 and C24 bile acids and their respective roles in liver injury and recovery.

## 5 Conclusion

In this study, we employed an integrated approach combining network analysis, molecular docking, molecular dynamics simulations, and experimental validation in a zebrafish chemical injury model to investigate the hepatoprotective effects of BBR against TAA-induced liver injury. Our results demonstrated that BBR effectively ameliorates TAA-induced hepatic lipid accumulation and bile acid dysregulation. Mechanistically, BBR restores homeostatic function to the dysregulated PPARγ signaling axis, modulating transcriptional programs toward hepatoprotective processes, including bile acid metabolism. These findings provide moleculnar evidence supporting the ethnopharmacological use of *C. chinensis* and identify potential therapeutic targets for liver injury, though further validation in metabolic NAFLD models is required.

## Data Availability

The original contributions presented in the study are included in the article/Supplementary Material, further inquiries can be directed to the corresponding authors.
